# Measuring Left Ventricular Volumes in Two-Dimensional Echocardiography Image Sequence Using Level-set Method for Automatic Detection of End-Diastole and End-systole Frames

**DOI:** 10.5812/cardiovascmed.6397

**Published:** 2013-02-24

**Authors:** Saeed Darvishi, Hamid Behnam, Majid Pouladian, Niloufar Samiei

**Affiliations:** 1 Faculty of Biomedical Engineering, Department of Biomedical Engineering, Science and Research Branch, Islamic Azad University, Tehran, IR Iran; 2 Department of the Electronic Engineering, Iran University of Science and Technology, Tehran, IR Iran; 3 Department of Biomedical Engineering, Science and Research Branch, Islamic Azad University, Tehran, IR Iran; 4 Echocardiography Research Center, Rajaie Cardiovascular Medical and Research Center, Tehran University of Medical Sciences, Tehran, IR Iran

**Keywords:** Echocardiography, Segmentation, Systole, Diastole, Ejection Fraction, Cardiac Output

## Abstract

**Background:**

Identifying End-Diastole (ED) and End-Systole (ES) frames is highly important in the process of evaluating cardiac function and measuring global parameters accurately, such as Ejection Fraction (EF), Cardiac Output (CO) and Stroke Volume.

**Objectives:**

The current study aimed to develop a new method based on measuring volume changes in Left Ventricle (LV) during cardiac cycle.

**Material and Methods:**

For this purpose, the Level Set method was used both in detecting endocardium border and quantifying cardiac function of all frames.

**Results:**

Demonstrating LV volumes displays ED and ES frames and the volumes used in calculating the required parameters.

**Conclusions:**

Since ES and ED frames exist in iso-volumic phases of the cardiac cycle with minimum and maximum values of LV volume signals, such peaks can be utilized in finding related frames.

## 1. Background

It has been acknowledged that accurate measurement of ventricular volumes (LV) and Ejection Fraction (EF) are of paramount importance and that is why recent clinical principles are based on volumetric and EF measures in diverse patient groups (1,2). Given that identifying ED and ES frames in 2D or 3D echocardiography image sequences are absolutely crucial for precise determination of a wide variety of variables along with their correlations used during the assessment of left ventricular function (3-9), the 2D echocardiography is the most commonly used approach for such measurements. 

To typify universal cardiac functions, a significant number of factors have been investigated to measure ES and ED volumes, SV, global EF ratio, CO and wall thickening, all of which are essential parameters to assess heart function (10-14). ED is often used as the landmark by which the timing of other events in the cardiac cycle is referred to (15-18). It is also noteworthy that detecting these frames is basically required for a number of post-processing techniques such as calculating 2D strain rate or color kinesis (19). The frame corresponding to the maximal cavity area achieved at the heart expansion phase is the End-Diastole Frame. Likewise, the frame corresponding to the minimal cavity area achieved at the contraction phase is the frame assumed to be the systole end (20). By and large, identifying End-Systole and End-Diastole Frames is still visually conducted by means of cardiac cycle movie and a trackball assist. Generally speaking, there are three ways to identify ED frames in ultrasound sequences:

- Electrocardiogram (ECG) R-wave
- Following mitral valve closure
- Maximal/minimal ventricular volumes and ES Frame selection after opening the mitral valve(10).

ED may also be visually identified by means of a vertical cursor in a Graphical User Interface (GUI) in particular. The best way (Gold Standard) is to identify ED manually, which is, however, time-consuming and laborious. To resolve this problem, numerous methods have been suggested to detect End-Systole Frames automatically based on 2D echocardiography sequences (21-27). Nevertheless, all such methods based on the first or second derivatives of the Left Ventricular Pressure (LVP), or the Electrocardiogram (ECG) identify ED indirectly. For instance, ED has been commonly known as a point where the first derivative of LVP rises above a certain threshold (27-31). However, these thresholds may not reckon the precise time of ED among incongruous patients at different age ranges who have dissimilar features, i. e. heart rates and cardiac loading conditions. 

Myocardial excitation−contraction holdup (16) may also affect accurate application of electrocardiogram R-wave (ECGR) peak to define ED (3,30). Being detected at the time of rapid mitral valve opening in early diastole, ES Frames(19) indicated by the intensity variation time curves within the cavity region, were measured in each pixel. Three landmarks of apex along with every angle of mitral annulus contribute to detection of this cavity. There has also been another method based on the left ventricular deformation during the cardiac cycle. Accordingly, the deformation curve is assessed by the correlation coefficients among the End-Diastolic image and subsequent images of a cardiac cycle. Then, the minimal correlation is adjusted with the End-Systolic image. The first setback of such methods is that an expert user is always in demand. Moreover, intervention or considerable differences are realized from the results of such methods by data identification. Furthermore, it is inevitable to determine the End-Diastolic Frame. With respect to the above-mentioned flaws, another approach has been recently suggested, i. e. using manifold learning to predict these frames automatically, although the evaluation of the method is not reliable (32).

## 2. Objectives

This paper aimed to introduce a new method to detect End-Diastole and End-Systole Frames of echocardiography image sequences by image segmentation automatically. An overview of cardiac-cycle physiology, as well as the description of Level Set Method is presented. Illustrative results concerning the image of two- chamber long axis echocardiography images will be discussed in Section 3, an efficient method to spot End-systole and End-Diastole Frames is also investigated. Lastly, the advantages and limitations of the study will be summarized in Section 4.

## 3. Materials and Methods

Accurate Left Ventricle (LV) segmentations during a cardiac cycle provide not only useful quantitative parameters, e. g. ejection fraction, but also qualitative information for certain heart conditions diagnosis. 

### 3.1. Cardiac Cycle

**Figure 1.**
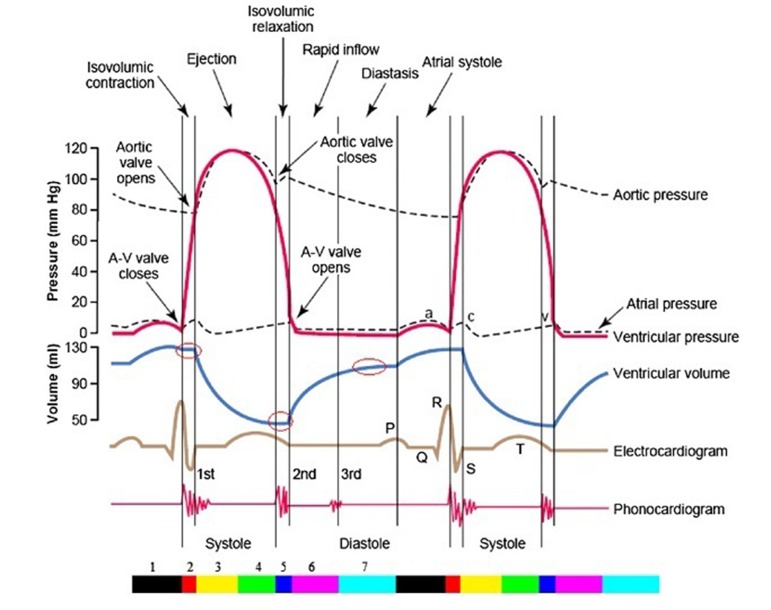
The entire two cardiac cycle diagram, which contains information on aortic, left ventricular and left atrial pressures, along with ventricular volume, heart sounds and the electrocardiogram. Three isovolumic intervals are emphasized by circles in the ventricular volume diagram. The color bar illustrates seven phases of the cardiac cycle (first phase: black, second: red, third: yellow, fourth: green, fifth: blue, sixth: magenta, seventh: cyan) (20)

A cardiac cycle encompasses every single event that occurs during a heartbeat. Figure 1 illustrates a typical cardiac cycle. This cycle is of two separate stages, i. e. diastole and systole (20). In order to analyze these stages comprehensively, a cardiac cycle is divided into seven phases (33). The first phase is linked with atrial contraction, initiated by the P-wave of the ECG as a result of electrical depolarization of atria. The second phase, isovolumetric contraction, begins with Atrio Ventricular (AV) valve closures and ends with the opening of the aortic and pulmonic valves. During this phase, the ventricular volume is at its maximum known as the End-Diastolic Volume (EDV). In the third phase, rapid ejection, blood flows into the aorta and pulmonary arteries rapidly. The fourth phase, i. e. reduced ejection, is the beginning of ventricular contraction. This phase is characterized by T- wave in ECG. The fifth phase is isovolumetric relaxation when the closure of aortic and pulmonic valves happens and successivly ends with the opening of AV valves. This volume is called End-Systolic Volume. The sixth phase is rapid filling, and the seventh phase is reduced filling as the ventricles continue to fill. On the other hand, there are two steps in a cardiac cycle which has minimum and maximum of ventricular volume diagram in Figure 1 (20).

### 3.2 Data acquisition

**Figure 2.**
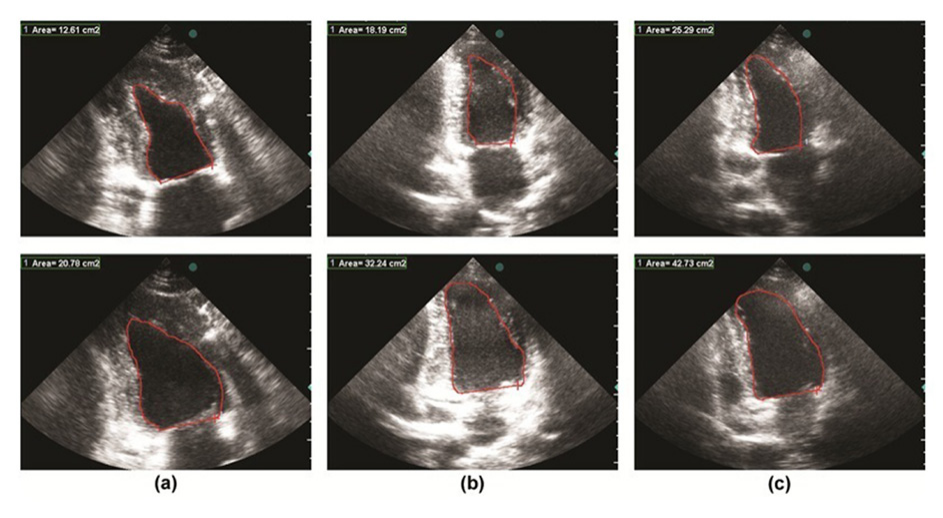
End-diastolic (top row) and corresponding end-systolic frames (bottom row) which have been extracted by the expert for three random cases: (a) case 1, (b) case 2 and (c) case 3.

The GE Vivid 7 ultrasound machinery provided the apical two-dimensional gray scale sequences of 44 volunteers, stored in AVI format including the ECG display, in which the two-chamber long axis views were used. Additionally, the sequences of three successive cycles were stored while separated cycles were identified by selecting the QRS complex onset. To evaluate the suggested method, the image sequences were visually analyzed by an experienced echo cardiologist and the End-Systolic and End-Diastolic Frames were visually determined, for each of the views. The whole procedure was approved by Medical Ethics Committee at Medical Sciences Department of Tehran University. The length of each image sequence was approximately two seconds including 20 to 25 frames (640×480 pixels). Due to the fact that the data were collected by different ultrasound machines, in different settings, and by different individuals, they had unlike levels of noise. Table 1 shows data collected by the software and expert analysis. To evaluate the suggested method, the image sequences were visually analyzed by experienced echocardiologists. As indicated in Figure 2, the End-Systolic and End-Diastolic Frames were visually marked. Then, EF, CO and SV were measured.

### 3.3. Level Set Method

**Figure 3.**
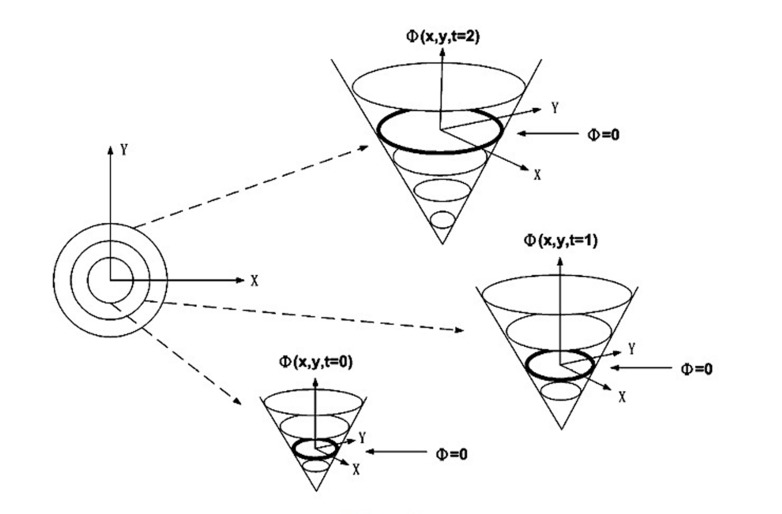
Level set formulation of front motion: the curve γ and the corresponding surface Φ(x,y,t) at different time t(36)

Having defined Formula (1) as in Figure 3, a group of closed contours is generated (34) by moving an initial contour towards its Euclidean normal inward vector N. (1)

c(p,t)={x (p, t), y (p, t)}

Considering scalar function F and speed of curve movement of curvature K, the equation will be as follows;

{ct(p, t)= F(k)Nc(p,0)= co(p)

In order to solve the partial differential equation, the Level Set Method was innovated by Sethian et,al (35,36) to signify the contour C(p,t), merely because the zero level set of a flat continuous scalar function Φ(x, y, t)is the level set function, where x, y ∈ R2. At any time t , a contour is calculated as follows:

c(p,t)={x,y|Φ(x, y, t)=0}

Concerning time and space, derivative of Φ(x, y, t) = 0 is obtained as follows:

{ Φt= -F(K)|∇Φ|Φ(co(p),0)=0

where ∇ is a gradient operator and |∇Φ| denotes the gradient norm. 

As a consequence, a group of moving curves C(p,t) are recognized corresponding with the group of evolving level set surfaces Φ(x, y, t). When Level Set Method is applied to find object boundary in images, a stoppage measure is multiplied by the speed function F(K) to discontinue the curve at the object boundary. This stoppage measure K1 is commonly defined in terms of gradient;

K1(x, y)= e-Eimage(x,y)

where Eimage(x,y)=|∇Gσ * I(x,y)| is the image energy calculated by convoluting the gradient of a Gaussian filter Gσ with image I(x, y). Thus, complete speed term of level set function will be as follows:

F(x,y)= F(K).K(x, y) = F(K).e-Eimage(x,y)1

**Figure 4.**
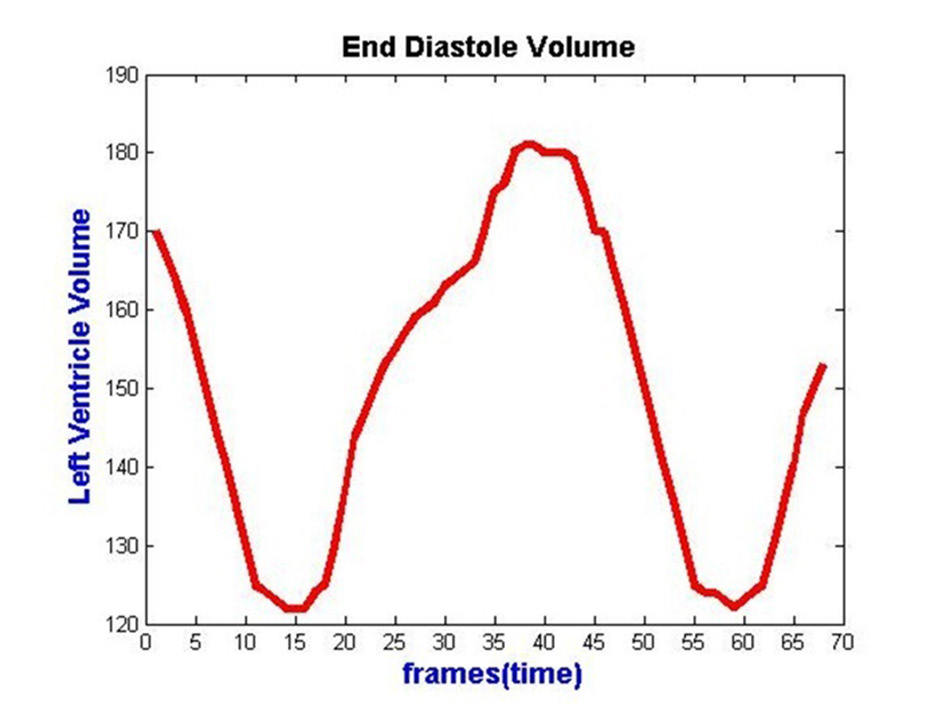
Left ventricle volume changes for all frames in cardiac cycle of a random case.

Once the curve moves towards the object boundary, the speed term is approximately zero, where the evolution process is stopped (37). Left Ventricle volume change during a random cardiac cycle is illustrated in Figure 4.

## 4. Results

**Figure 5.**
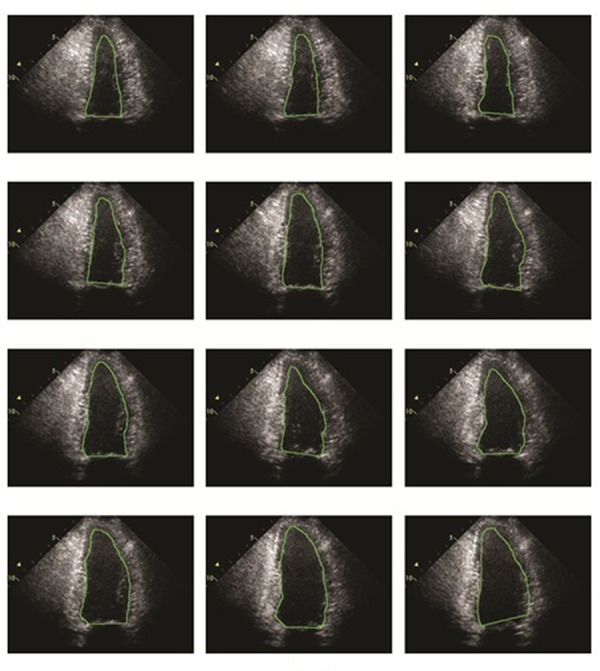
This picture shows the results of our proposed method for one cardiac cycle for a random case in the heart contraction and relaxation period

An algorithm was applied in MATLAB 7 (MathWorks, Natick, Massachusetts, United States). 6 to evaluate Left Ventricular function. Consequently, the impacts of the suggested method on a huge set of cardiac images were proven. It has been observed that such a method provides far more accurate segmentations compared with other approaches, especially once the observed data are of limited quality. In addition, figuring out how LV evolves throughout an entire cardiac cycle permits physicians to determine the health of myocardial muscles. Segmented LV boundaries can also be useful for further quantitative analyses. The results of the suggested method were compared with those of an echocardiographic expert. To implement the suggested method on each case, three cardiac cycles were considered. Figure 5 illustrates the results of the study during the heart contraction and relaxation period, respectively. As observed, the extracted contours are all similar to the ground truth. 

In physiology, ejection fraction represents the volumetric fraction of blood pumped out of the ventricle with every heartbeat (38). 

Stroke Volume (SV) = EDV – ESV

Ejection Fraction (EF) = (SV / EDV) × 100%

Cardiac Output (CO) = SV × HR

Heart Rate (HR)= BPM (Beats Per Minute)

**Figure 6.**
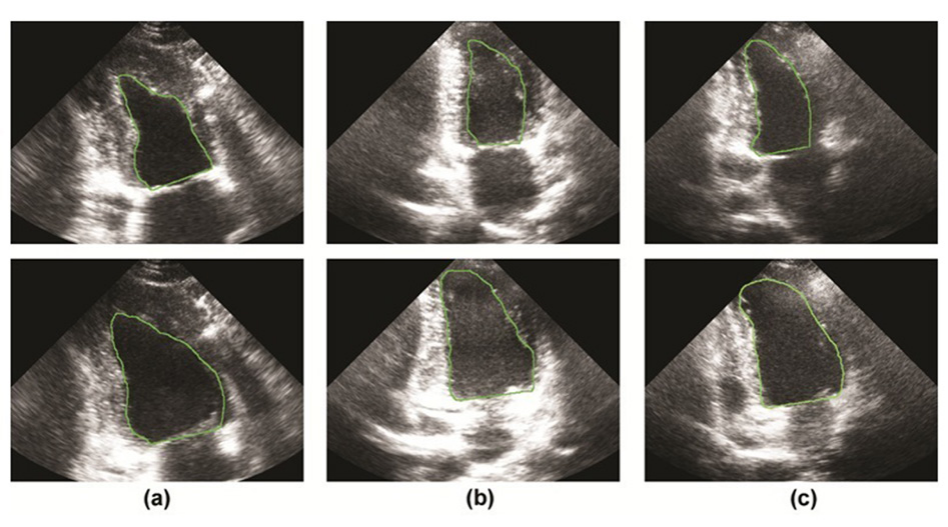
End-diastolic (top row) and corresponding end-systolic frames (bottom row) which have been extracted by the proposed method for three random cases: (a) case 1, (b) case 2 and (c) case 3.

To assess the accuracy of results, a number of metrics are applied, 

Table 1 displays a number of statistical results between visual reading and the suggested method for EF Parameter of all cases. Figure 6 shows the End-Systole and End-Diastole Frames extracted by the suggested method for three cases. 

**Table 1.** Statistical measurements for EF percent for all 44 cases

MAE ^a^	MSE ^a^	MRE ^a^	RMSE ^a^
1. 4967	3. 7038	0. 0246	1. 9245

^a^ Abbreviations: MAE: Mean Absolute Error, MRE: Mean Relation Error, MSE: Mean Squared Error, RMSE: Root Mean Squered Error

**Figure 7.**
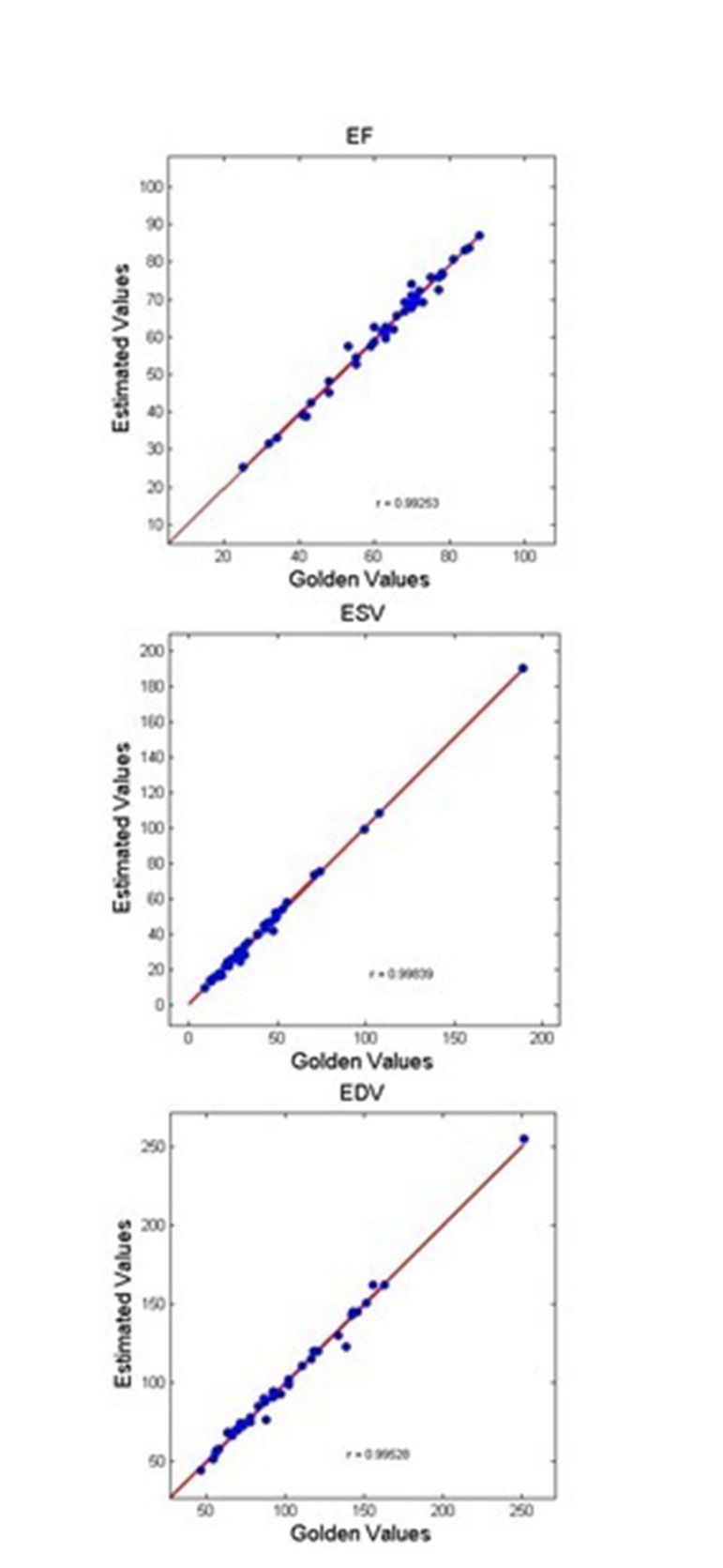
Linear Regression Between Estimated and Ground Truth EF, ESV, EDV for all Examined Cases

The results obtained from this method were validated with those of experienced echo cardiologist (Golden Standard) on 44 volunteers. To conduct statistical analysis, P was calculated using EF obtained with the reference to visual reading, and that of automatically estimated using the suggested method. The results indicated the significance of the presented method (P = 0. 8057). In Figure 7and8 , linear regression and Bland-Altman figures for EF ,EDV and ESV have shown respectively. 

**Figure 8.**
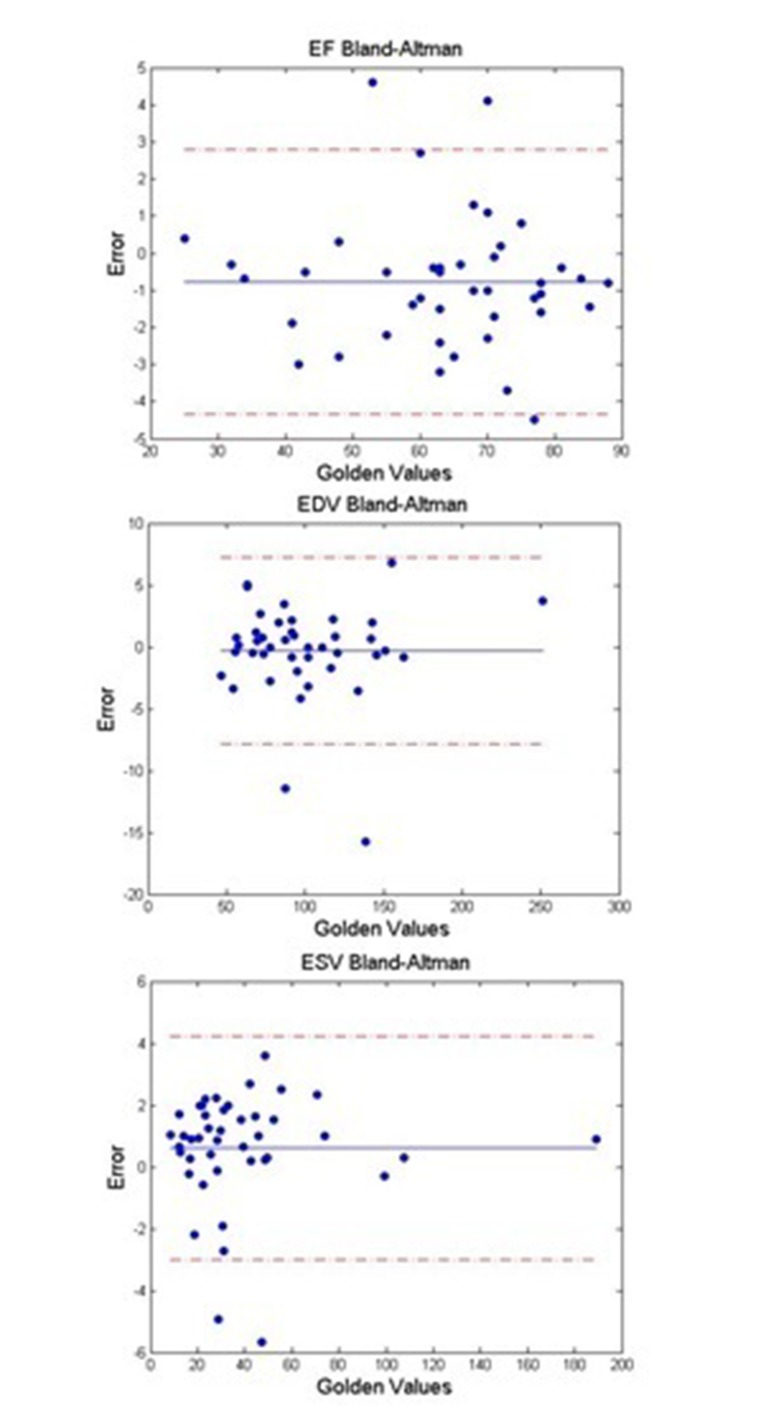
Bland-Altman plot to show the agreements Between Estimated and Ground Truth EF, ESV, EDV for all Examined Cases

## 5. Discussion

The results of the carried out experiments and their comparison with those of the ground truth, indicated that the suggested method effectively prevented automatic detection of ED and ES Frames and contributed to more improved detection in comparison with other approaches without imposing the burden of calculation. More interestingly, this method is not only cost effective, but also effortlessly applied in such a way that any ambiguity resulted from the human assessment is fortunately overcome. Consequently, this method has its potentials and efficiency for the intended tasks. 

Most cardiac ultrasound imaging systems have a built-in ECG recording system to assist the cardiologist find the End-Systole and End-Diastole Frames. Otherwise, the reliable detection of such frames becomes a demanding issue. Based on this method, what is required is the ultrasound image and, thus, there is no need to have the ECG Capturing System together with ultrasound imaging. Due to noisy temperament of echocardiography images, the assessment of cardiac function via other analogous methods is truly problematic, whereas the suggested method is not sensitive to noise. Similarly, the results indicate that the suggested method is applicable for normal and abnormal cases. For that reason, the researchers are to develop an approach based on 3D echocardiography that lately has been warmly welcomed by the Cardiology Community. 

The results of the suggested method demonstrate its strong potential to analyze data in sets of echocardiography images. The researchers maintain that the dedicated segmentation approaches will open a new horizon to analyzing the medical images and echocardiography images, in particular.
